# Antioxidant Supplementation Alleviates Mercury-Induced Cytotoxicity and Restores the Implantation-Related Functions of Primary Human Endometrial Cells

**DOI:** 10.3390/ijms24108799

**Published:** 2023-05-15

**Authors:** Andrea Palomar, Alicia Quiñonero, Yassmin Medina-Laver, Roberto Gonzalez-Martin, Silvia Pérez-Debén, Pilar Alama, Francisco Domínguez

**Affiliations:** 1 Reproductive Medicine Research Group, IVI Foundation—IIS La Fe Health Research Institute, 46026 Valencia, Spain; andrea.palomar@ivirma.com (A.P.); alicia.quinonero@ivirma.com (A.Q.); yassmin.medina@ivirma.com (Y.M.-L.); roberto.gonzalez@ivirma.com (R.G.-M.); 2 Biomedical Research Institute INCLIVA, 46010 Valencia, Spain; silviapede86@gmail.com; 3 Department of Gynecology, IVIRMA-Valencia, 46015 Valencia, Spain; pilar.alama@ivirma.com

**Keywords:** mercury (Hg), primary endometrial epithelial cells (hEnEC), primary decidual endometrial stromal cells (d-hEnSC), trophoblastic outgrowth, oxidative stress (OS), infertility, spheroid, co-culture

## Abstract

Mercury (Hg) cytotoxicity, which is largely mediated through oxidative stress (OS), can be relieved with antioxidants. Thus, we aimed to study the effects of Hg alone or in combination with 5 nM N-Acetyl-L-cysteine (NAC) on the primary endometrial cells’ viability and function. Primary human endometrial epithelial cells (hEnEC) and stromal cells (hEnSC) were isolated from 44 endometrial biopsies obtained from healthy donors. The viability of treated endometrial and JEG-3 trophoblast cells was evaluated via tetrazolium salt metabolism. Cell death and DNA integrity were quantified following annexin V and TUNEL staining, while the reactive oxygen species (ROS) levels were quantified following DCFDA staining. Decidualization was assessed through secreted prolactin and the insulin-like growth factor-binding protein 1 (IGFBP1) in cultured media. JEG-3 spheroids were co-cultured with the hEnEC and decidual hEnSC to assess trophoblast adhesion and outgrowth on the decidual stroma, respectively. Hg compromised cell viability and amplified ROS production in trophoblast and endometrial cells and exacerbated cell death and DNA damage in trophoblast cells, impairing trophoblast adhesion and outgrowth. NAC supplementation significantly restored cell viability, trophoblast adhesion, and outgrowth. As these effects were accompanied by the significant decline in ROS production, our findings originally describe how implantation-related endometrial cell functions are restored in Hg-treated primary human endometrial co-cultures by antioxidant supplementation.

## 1. Introduction

Successful embryo implantation is a complex process that is tightly regulated by hormones and depends heavily on correct endometrial function [1,2]. As such, disruptions in endometrial features caused by lifestyle, diet, or environmental contaminants can negatively impact reproductive health and fertility. Indeed, exposure to endocrine disruptors and/or other environmental pollutants can alter endometrial cell function and jeopardize reproductive success [3].

Humans are exposed to mercury (Hg), a naturally ubiquitous divalent metal [4], mainly through dietary sources [5,6]. Independent studies have shown that Hg exposure has a deleterious effect on reproductive physiology [7,8,9], and gestational exposure may negatively impact fetal development [10]. In patients undergoing in vitro fertilization (IVF), Hg exposure was associated with decreased ovarian response to gonadotropins, diminished follicle count after ovarian stimulation, fewer oocytes recovered, delayed time to pregnancy, and an increased risk of miscarriage [11]. These findings were corroborated by two studies that reported higher whole-blood Hg levels in infertile women (12,13), and a large association study that determined a non-linear association between the levels of this heavy metal in blood and infertility [12].

Despite the rising concerns regarding the effect of Hg exposure on human fertility, most studies published to date were performed in animal models or cell lines [13,14,15,16,17], with few addressing the direct impact of this heavy metal on primary human cells. Our group recently demonstrated that Hg treatment impairs decidualization [18]; however additional studies evaluating human trophoblast adhesion to the endometrial epithelium and subsequent outgrowth will contribute to the understanding of early embryo development, placentation, and the achievement of pregnancy [19].

Heavy metals can cause tissue damage by promoting oxidative stress (OS), in part by decreasing the activity of several antioxidant enzymes (e.g., superoxide dismutase, glutathione, glutathione reductase, or catalase) [15,16,17,20,21]. Compounds of different natures, such as essential trace elements zinc [22] and selenium [23], as well as phenolic compounds, such as curcumin [17], butylated hydroxytoluene (BHT) [24], and derivates from lipoic acid [25], share a common mechanism to ameliorate Hg-induced cytotoxicity throughout the activation of antioxidant defense genes included in the Nrf2/ARE pathway. Considering that these antioxidant compounds alleviated Hg-induced damage in rat cell lines and primary cultures [17,22,23,24,25], the aim of this study was to characterize the extent of Hg-induced damage on primary endometrial cells’ viability and function and confirm whether antioxidant therapy reverses this endometrial damage. To our knowledge, this is the first record of in vitro co-culture models using primary human endometrial epithelial (hEnEC) or stromal cells (hEnSC) and JEG-3 cells within this context, to more accurately reflect the cellular response to exogenous Hg-induced stress, with respect to immortalized cell lines [14]. Further, the inclusion of co-culture in vitro models using JEG-3 trophoblastic spheroids and primary endometrial cells allows us to study the effect of Hg and antioxidant supplementation in both types of cells and how to modulate the adhesion and invasion processes.

## 2. Results

### 2.1. Hg Treatment Compromises the Viability of Human Primary Epithelial Cells (hEnEC) by Inducing Oxidative Stress (OS)

**Figure 1:**
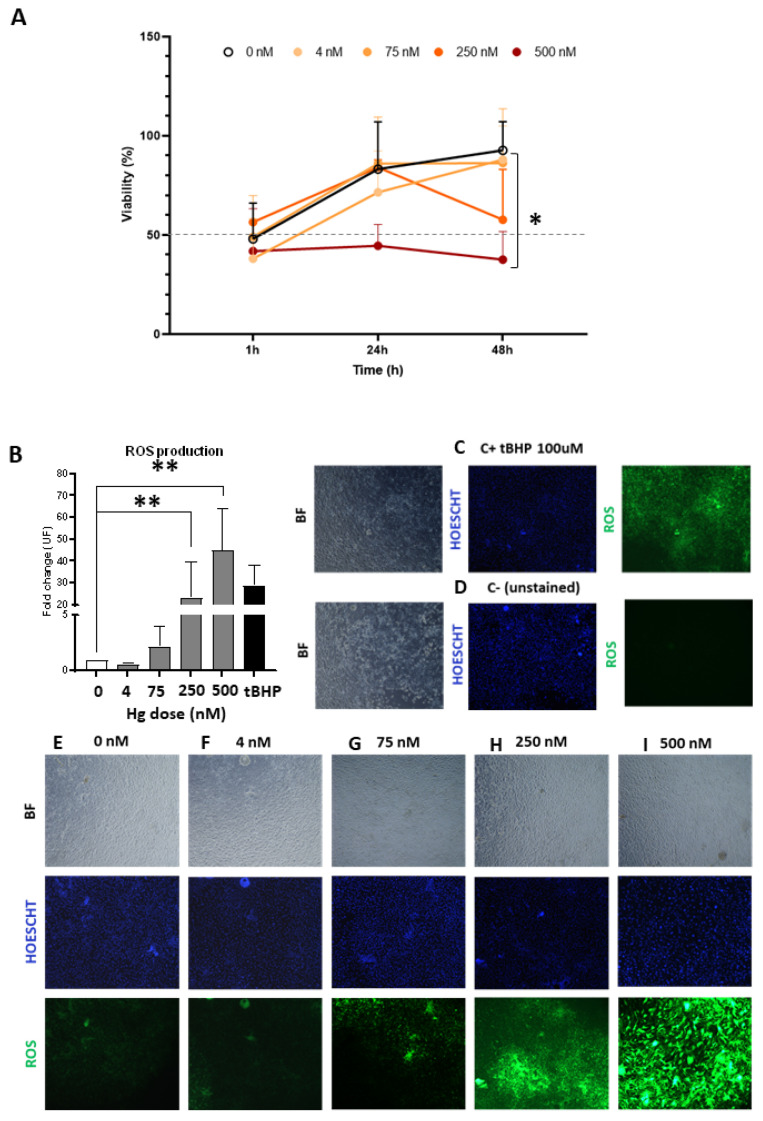
Cell viability and reactive oxygen species (ROS) production of primary human endometrial epithelial cells (hEnEC) after exposure to 0, 4, 75, 250, or 500 nM of mercury (Hg). (**A**) Cell viability was assessed after treatment with Hg, using modified tetrazolium salt (MTS) assays. * *p* < 0.05. (**B**) ROS were quantified by analysis of dichlorofluorescin diacetate (DCFDA). The fold change in fluorescence was calculated relative to the untreated group (0 nM). Data are presented as an overall mean ± standard deviation (SD). ** *p* < 0.001. Photomicrographs of ROS production in hEnEC monolayers following incubation with a ROS inductor (100 μM tert-Butyl hydroperoxide (tBHP); positive control (C+)); (**C**), no fluorogenic dye (negative control [C−]); (**D**), or 0, 4, 75, 250, or 500 nM of Hg (**E**–**I**). Images were captured at 200× magnification using brightfield (BF) and fluorescent microscopy to identify cells (nuclei stained blue with Hoescht) with ROS activity (stained green).

To assess the Hg-induced damage in primary hEnEC cultures, we evaluated cell viability and reactive oxygen species (ROS) production as a marker for OS. Within the first 24 h of treatment with a Hg dose ≤ 250 nM, a gradual increase in the epithelial cells’ viability was observed, with no significant differences between treatments (Figure 1A). On the contrary, the viability of primary hEnEC treated with 500 nM of Hg remained relatively stable within the first 24 h, but was significantly lower than the untreated controls at the 48 h time point (*p* < 0.05), exhibiting a mean downturn in cell viability below 50% compared to the control.

The 250 and 500 nM doses, respectively, induced significant 25-fold (*p* < 0.01) and 45-fold (*p* < 0.01) increases in hEnEC ROS production compared to the untreated controls (Figure 1B), while lower doses showed no significant changes in the ROS production (Figure 1B,E–G). These findings were supported by the enhanced ROS activity observed by fluorescence imaging in hEnEC monolayers treated with 250 nM (Figure 1H) and 500 nM of Hg (Figure 1I), with respect to lower doses (Figure 1E–G). Notably, the highest ROS levels observed with 500 nM of Hg treatment surpassed the ROS levels of the positive control (100 uM tBHP; Figure 1B).

### 2.2. Decidual Function Is Unaffected by Withdrawing or Maintaining a Mild Mercury (Hg) Treatment during Decidualization, Regardless of Prior Metal Treatment

**Figure 2:**
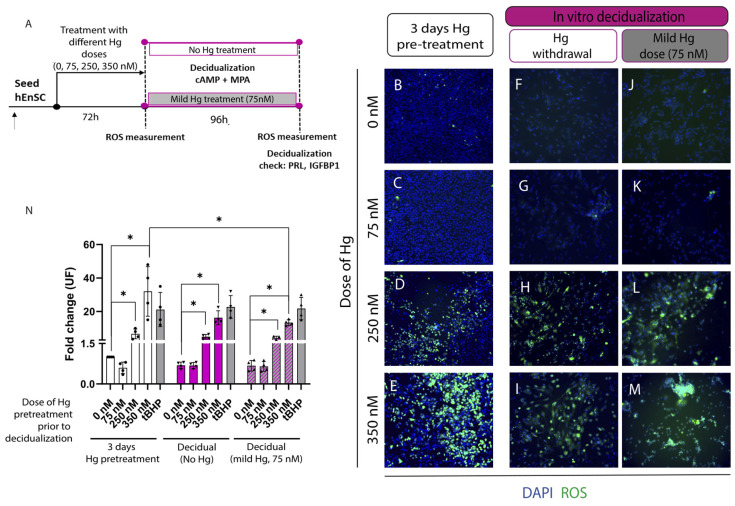
Oxidative stress (OS) of primary human endometrial stromal cells (hEnSC) treated with mercury (Hg) prior to and during in vitro decidualization. (**A**) Schematic workflow. Photomicrographs of reactive oxygen species (ROS) production in hEnSC, following pretreatment with 0, 75, 250, or 350 nM of Hg, prior to decidualization (**B**–**E**), and after in vitro decidualization with no (**F**–**I**) or mild (75 nM) metal stimulus (**J**–**M**). Images were captured at 200× magnification. (**N**) ROS were quantified by dichlorofluorescin diacetate (DCFDA) staininng for each condition and positive control (100 μM tert-Butyl hydroperoxide (tBHP)). The fold change was calculated with respect to the untreated group (0 nM). Data are presented as the overall mean fold change ± standard deviation (SD). * *p*-value < 0.05.

ROS production was measured in primary cultures of hEnSC pretreated with 0, 75, 250, or 350 nM of Hg for 72 h and in decidual primary human endometrial stromal cells (d-hEnSC) that received no or mild (75 nM) Hg treatment during decidualization to track variations in Hg-induced OS (Figure 2A). Treatment with high doses of this heavy metal (250–350 nM) for 72 h significantly augmented the ROS levels in primary hEnSC compared to the untreated controls (*p* < 0.05; Figure 2B,D,E,N). No significant differences in the ROS production were observed with milder doses of Hg (75 nM) for 72 h (Figure 2C,N). After in vitro decidualization (with or without 75 nM of Hg), d-hEnSC pretreated with 250–350 nM of Hg (Figure 2H,I,L,M) showed ROS levels compared to d-hEnSC pretreated with 0–75 nM of Hg (Figure 2F,G,J,K). The ROS in d-hEnSC pretreated with high Hg doses (250–350 nM) remained significantly higher than untreated d-hEnSC, regardless of there being no or mild metal stimuli during decidualization (*p* < 0.05; Figure 2N).

Next, the secretion of decidual markers, prolactin (PRL) and insulin-like growth factor-binding protein 1 (IGFBP1) was wuantified in the culture media to evaluate the efficacy of decidualization in primary cultures of hEnSC (Supplementary Figure S2A). Interestingly, ROS production in primary hEnSC pretreated with 350 nM of Hg was significantly reduced following decidualization (*p* < 0.05, Figure 2N). Accordingly, the rise in ROS activity in primary hEnSC treated with 350 nM of Hg for 72 h (Figure 2E) was mitigated with decidualization, regardless of there being no (Figure 2I) or mild (75 nM) metal stimulus (Figure 2M). Further, after comparing these decidual conditions, we found no significant differences in terms of the ROS activity (Figure 2N) or secretion of PRL and IGFBP1 (Supplementary Figure S2B,C), suggesting that the decidual function was unaffected by the complete withdrawal or maintenance of a mild metal dose during in vitro decidualization regimen.

### 2.3. Antioxidant Supplementation Enhances Cell Viability by Alleviating OS in Primary hEnEC Exposed to Hg

**Figure 3:**
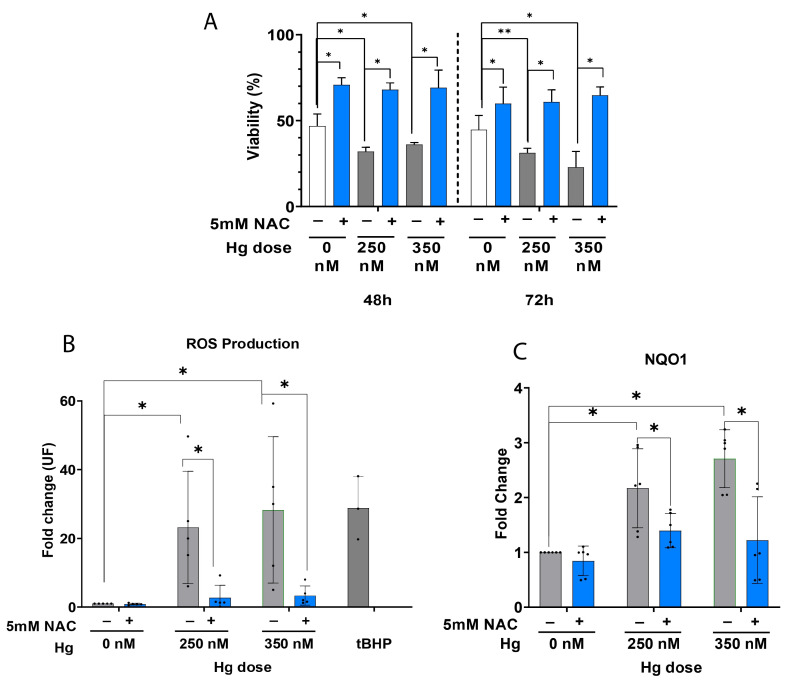
Oxidative stress (OS) in human endometrial epithelial cells (hEnEC) exposed to 0, 250, or 350 nM of mercury (Hg) alone or in combination with 5 mM N-Acetyl-L-cysteine (NAC). (**A**) Viability was determined by modified tetrazolium salt (MTS) assay. (**B**) Reactive oxygen species (ROS) were quantified by dichlorofluorescin diacetate (DCFDA) staining following 24 h treatment. hEnEC treated with 100 μM tert-Butyl hydroperoxide (tBHP) served as a positive control. (**C**) mRNA expression of *NQO1* after 72 h treatment. In both (**B**,**C**), data are presented as the overall mean fold change values ± standard deviation (SD), calculated with respect to the untreated group (0 nM). * *p*-value < 0.05. ** *p*-value < 0.001.

To evaluate the extent to which antioxidant supplementation 5 mM N-Acetyl-L-cysteine (NAC) can reverse Hg-induced oxidative damage and improve cell viability in primary hEnEC cultures, we measured ROS production and *NQO1* expression. NAC supplementation significantly enhanced the cell viability of hEnEC treated with 250 or 350 nM of Hg for 48 h or 72 h, with respect to the untreated (0 nM) control (*p* < 0.05; Figure 3A). This finding was supported by the significant NAC-mediated decline of the significantly elevated ROS levels we observed in the primary hEnEC after the first 24 h of culture with 250 nM and 350 nM of Hg, compared to the untreated (0 nM) controls (*p* < 0.05; Figure 3B). Similarly, *NQO1* was significantly upregulated in primary hEnEC treated with 250–350 nM HgCl_2_ (*p* < 0.05; Figure 3C), but antioxidant supplementation significantly repressed the expression (*p* < 0.05), restoring the mRNA levels to near-control (Figure 3C).

### 2.4. Antioxidant Supplementation Enhances Cell Survival by Alleviating Oxidative Stress (OS), Reducing Apoptosis and Protecting DNA Integrity in JEG-3 Trophoblast Cells Exposed to Mercury (Hg)

**Figure 4:**
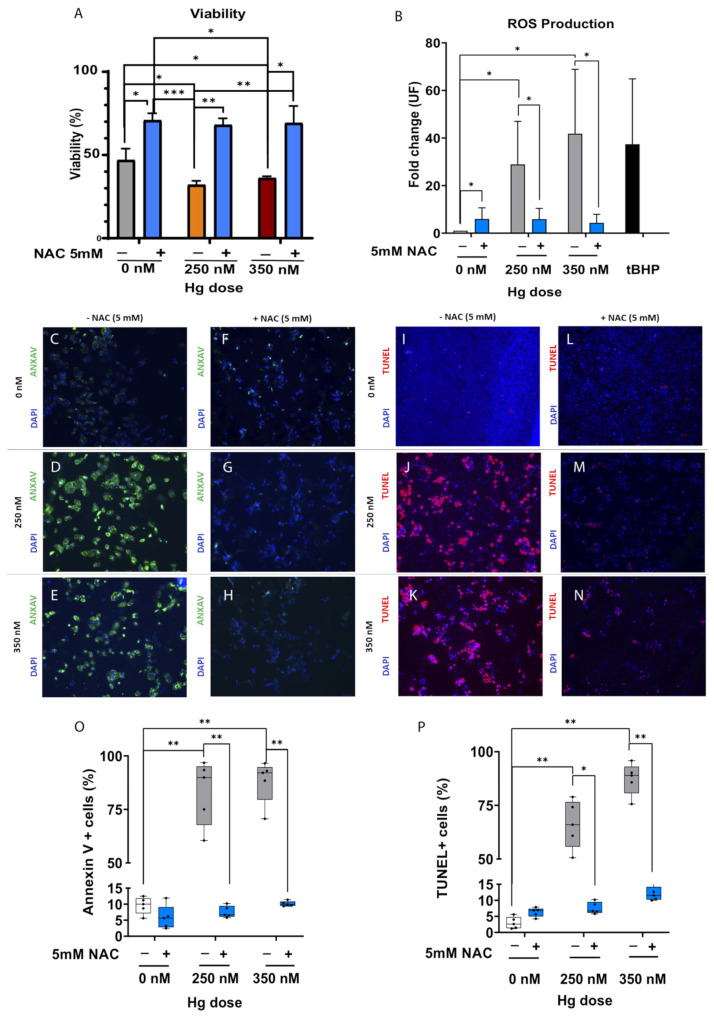
Antioxidant treatment improves viability and alleviates oxidative stress (OS) in JEG-3 trophoblast cells exposed to 0, 250, or 350 nM of mercury (Hg) alone or in combination with 5 mM N-Acetyl-L-cysteine (NAC) for 24 h or 48 h. (**A**) Viability of JEG-3 cells was assessed by modified tetrazolium salt (MTS) assays. (**B**) Reactive oxygen species (ROS) were quantified by dichlorofluorescin diacetate (DCFDA) following 48 h of treatment. JEG-3 cells treated with 100 μM tert-Butyl hydroperoxide (tBHP) served as a positive control. Data are presented as the overall mean fold change ± standard deviation (SD), calculated with respect to the untreated group (0 nM). Photomicrographs of (**C**–**H**) Annexin V (ANXA V) immunostaining (green) and (**I**–**N**) TUNEL staining (red) in JEG-3 cells (counterstained blue with DAPI). Images were captured at 200× magnification. The mean proportions of ANXA V (**O**) and TUNEL-positive cells (**P**) ± SD were calculated with respect to the total number of DAPI-positive nuclei in three different fields of view. * *p*-value < 0.05. ** *p*-value < 0.01. *** *p*-value < 0.001.

To evaluate the induced damage of this heavy metal on JEG-3 trophoblast cells, cell viability was assessed using a modified tetrazolium salt (MTS) assay, apoptosis via annexin V (ANXA V) expression, and double-strand DNA damage using TUNEL. In parallel, ROS production was quantified to measure improvements in OS when Hg-treated JEG-3 trophoblast cells were supplemented with 5 nM NAC. JEG-3 cell viability was significantly compromised following treatment with 250 or 350 nM of Hg for 48 h (*p* < 0.05; Figure 4A). However, supplementation with 5 mM NAC not only significantly enhanced the survival of the untreated controls (*p* < 0.05), but it also rescued the survival of JEG-3 cells treated with 250 nM (*p* < 0.01) and 350 nM of Hg (*p* < 0.05; Figure 4A). Accordingly, there was a significant boost in the ROS production when JEG-3 trophoblast cells were treated with 250 or 350 nM of Hg (*p* < 0.05; Figure 4B); however, this oxidative stress was alleviated with antioxidant supplementation (*p* < 0.05; Figure 4B). These findings were supported by the significant amplification of apoptotic cells following 250–350 nM of Hg treatment (*p* < 0.01; Figure 4C–E,O), and the antioxidant-mediated rescue to near-control proportions (*p* < 0.01; Figure 4F–H,O). Finally, despite treatment with 250–350 nM of Hg producing extensive DNA damage (*p* < 0.01; Figure 4I–K,P), antioxidant supplementation significantly restored DNA integrity in JEG-3 cells treated with 250 nM (*p* < 0.05; Figure 4M,P) or 350 nM of Hg (*p* < 0.01; Figure 4L–N,P).

### 2.5. Antioxidant Supplementation Rescues Trophoblast Adhesion and Outgrowth following Mercury-Induced Damage

**Figure 5:**
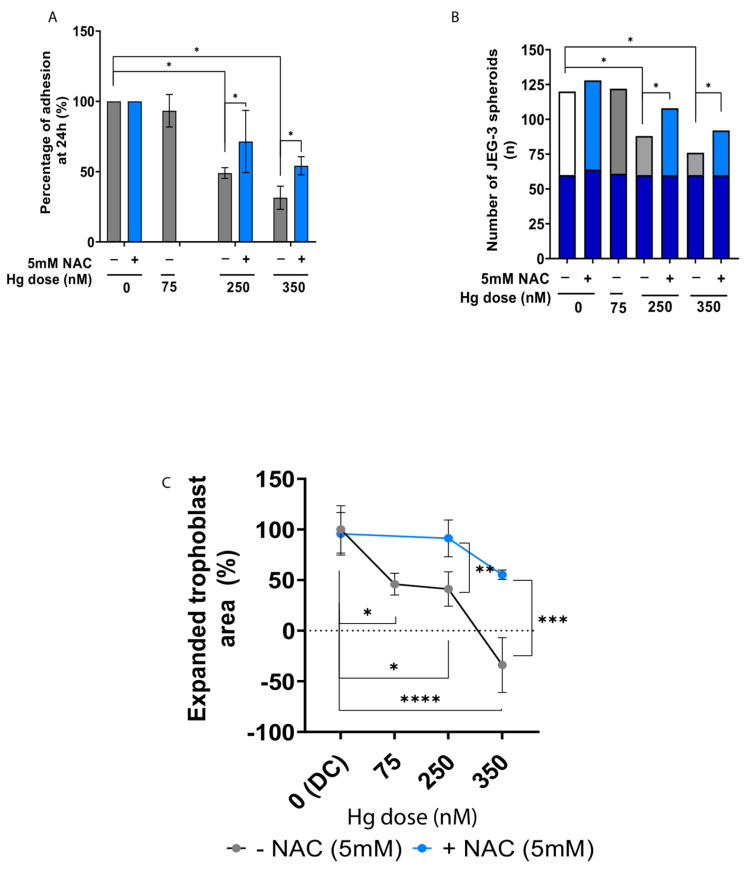
Evaluation of JEG-3 trophoblastic spheroid adhesion and outgrowth into primary decidual human endometrial stromal cells (d-hEnSC) pretreated with mercury (Hg) alone or in combination with 5 mM N-Acetyl-L-cysteine (NAC). (**A**) Percentage of adhered JEG-3 trophoblastic spheroids on primary human endometrial epithelial cells (hEnEC) pre-treated with Hg alone (grey bars) or in combination with NAC (blue bars). (**B**) Number of JEG-3 trophoblastic spheroids seeded (dark blue lower portion of the bars) and attached (upper portion of the bars) after 24 h co-culture on primary hEnEC pre-treated with Hg alone (grey portion of the bars) or in combination with NAC (light blue portion of the bars). (**C**) Expanded trophoblast area of JEG-3 co-cultured for 48 h with d-hEnSC pre-treated with different Hg doses, either supplemented or not with NAC. Expanded trophoblast area was expressed as a percentage calculated with respect to the untreated condition. Data correspond to mean values ± standard deviation (SD). Note: the groups treated with 75 nM of Hg in (**A**–**C**) were not supplemented with NAC as previous outcomes on cell viability did not reflect significant impairment when cells were treated with this Hg concentration. Thus, NAC treatment was only considered in the two highest doses of Hg and untreated control. * *p*-value < 0.05. ** *p*-value < 0.01. *** *p*-value < 0.001. **** *p*-value < 0.0001. DC: decidual unexposed control.

JEG-3 spheroids were co-cultured with primary hEnEC monolayers that were pretreated with Hg alone or in combination with antioxidant supplementation (5 mM NAC) to assess trophoblastic spheroid adhesion. This model evidenced a significantly reduced capacity of the JEG-3 spheroids to adhere to the primary hEnEC monolayers treated with 250 or 350 nM of Hg (*p* < 0.05; Figure 5A) after 24 h of co-culture. Consequently, significantly fewer spheroids were attached to the primary hEnEC monolayers exposed to 250 or 350 nM of Hg compared to 0 or 75 nM of Hg (*p* < 0.05; Figure 5C). However, while Hg treatment (250–350 nM) significantly compromised the spheroids’ attachment compared to the untreated controls (*p* < 0.05), antioxidant supplementation largely promoted spheroid attachment at 24 h (*p* < 0.05), restoring levels to near-control (Figure 5A,B).

To model trophoblastic outgrowth into endometrial stroma, JEG-3 trophoblastic spheroids were co-cultured with primary d-hEnSC. Treatment with 75, 250, or 350 nM of Hg did not significantly affect decidualization of the hEnSC, as measured by similar PRL and IGFBP1 secretion with respect to the untreated (0 nM) group (Supplementary Figure S2E,F). All doses significantly hindered trophoblast outgrowth on Hg-treated d-hEnSC (*p* < 0.05 for 75 nM, *p* < 0.05 for 250 nM, and *p* < 0.0001 for 350 nM) compared to the untreated (0 nM) controls (Figure 5C). Further, trophoblast outgrowth was significantly reduced between the highest (350 nM) and mild Hg doses (75 nM; *p* < 0.05; Figure 5C). Finally, the addition of NAC significantly improved JEG-3 trophoblast outgrowth in co-cultures treated with 250, or 350 nM of Hg (*p* < 0.01 and *p* < 0.001, respectively; Figure 5C and Supplementary Figure S3A–C), while PRL and IGFBP1 production remained unaffected by antioxidant supplementation (Supplementary Figure S2E,F).

## 3. Discussion

The effects of Hg have been studied in multiple cell lines and animal models [22,23,24,25,26,27,28,29,30,31,32,33,34,35,36,37,38,39,40]; however, evidence regarding primary cells remains scarce. To address this research gap within the context of female reproduction, the extent of Hg-induced damage on endometrial cells’ viability and function using primary cultures of human endometrial cells were investigated in this research. This study highlighted that antioxidant supplementation alleviates OS, a key driver of this heavy metal cytotoxicity, restoring human endometrial cell functions related to implantation.

Our results demonstrated that Hg treatment causes a dose-dependent decrease in the viability of primary hEnEC. This finding complements the dose-dependent decrease reported in primary hEnSC viability [18] and supports the reduction in primary endometrial cell viability observed following treatment with other heavy metals such as copper [41]. Hg-induced toxicity is associated with enhanced cell death, which is evidenced by apoptosis in human and non-human cell lines [22,23,24,25,30,40] and autophagy in rat kidney cell lines [36], corroborating the plunge in primary hEnEC survival observed in this study.

Regarding the effect of heavy metals on endometrial function, prior evidence confirmed that copper [41] and Hg [18] interfere with the decidualization process by impeding primary hEnSC from acquiring a decidual phenotype. Interestingly, our current findings suggest that, regardless of pre-decidualization Hg exposure, neither withdrawing nor maintaining a mild Hg exposure during decidualization affects PRL and IGFBP1 levels. Reducing the exposure to this metal prior to inducing in vitro decidualization restored decidual function in primary cultures of hEnSC, which is in contrast to the effect observed when high Hg doses (>250 nM) persisted during in vitro decidualization [18]. Combined with the slight reduction in ROS production that we observed when the pretreated hEnSC were decidualized (with no or mild metal stimulus), these findings suggest that Hg-induced damage may be reversed when Hg is depleted.

Hg-induced cytotoxicity, which in this study, was evidenced by the impaired viability of primary hEnEC, is postulated to be driven by the substantial OS experienced by cells exposed to high Hg concentrations (>250 nM). This negative correlation between OS and cell survival is supported by similar findings reported in primary cultures of hEnSC [18], studies implying that OS mediates the toxicity of Hg [42] and other heavy metals such as arsenic [43], as well as studies in *Drosophila melanogaster* suggesting that heavy metal-mediated germline alterations could be due to OS [21]. Indeed, other groups agreed that inorganic [15,20] and organic [16,17] Hg-induced damage relies on the production of OS. Although Hg treatment was linked to OS in various cell lines [15,27,29,31,35,36], the doses of Hg used in cell lines and animal models are generally much higher than the Hg doses considered in this study because primary cells cannot tolerate such doses. Thus, we acknowledge that the cytotoxicity we observed in our primary human endometrial cells could be intensified with strong Hg exposure.

In accordance with previous evidence coinciding with an exacerbated OS response produced by Hg treatment, our findings support that Hg-induced cytotoxicity can be mitigated by antioxidant supplementation. The NAC-mediated reduction in ROS was also reflected by the repression of *NQO1* expression (Figure 3). *NQO1* plays a role in cell response to OS, and its expression is regulated by the *Nrf2-Keap* pathway. The nuclear factor erythroid 2 (*Nrf2*) is a key regulator of cellular defenses against oxidative, electrophilic, and environmental stress. In turn, *Nrf2* is regulated by Kelch-type ECH-associated protein 1 (*Keap1*), an adaptor subunit of the Cullin 3-based E3 ubiquitin ligase, which acts as a sensor protein for oxidative and electrophilic stresses. Thus, *Nrf2-Keap* signaling activates the transcription of OS-related genes, including *HO-1* and *NQO1*, among others [16]. Therefore, we reasoned that the decrease in *NQO1* expression observed when primary hEnEC were exposed to 250–350 nM of Hg and 5 mM NAC could help restore endometrial cell viability and function.

While previous research explored how treatment with heavy metals, including Hg, interfered with decidualization and the migration of primary hEnEC [18,41], our study provided a more in-depth characterization of how endometrial functions, particularly trophoblast adhesion and outgrowth on decidual stroma, are altered by Hg exposure. In this regard, the inclusion of co-cultures of primary endometrial cells and trophoblastic JEG-3 spheroids aims to mimic the interactions between trophoblast cells and endometrial epithelium and stroma occurring in vivo during the adhesion and invasion phases of human implantation. Our co-culture model corroborated previous findings using other heavy metals [41], with Hg treatment (>250 nM) preventing trophoblast adhesion to primary epithelial cells. The reduced adhesion in Hg-treated co-cultures may reflect a lack of adhesion proteins (e.g., cadherin-3) in trophoblast cells, as previously reported in the HTR8/SV-neo trophoblast cell line [44]. Nevertheless, we found that, at least within the context of human endometrial functions, metal-induced damage can be reversed by antioxidant therapy, since antioxidant supplementation effectively restored adhesion rates in co-cultures treated with high doses of Hg (>250 nM). Moreover, strong Hg exposure (>250 nM) substantially affected trophoblastic outgrowth into the decidual stroma, shedding light on how Hg impairs endometrial functions in humans and supporting the limited evidence in animal models demonstrating that metal treatment alters male rat fertility and ovarian function in *Drosophila melanogaster* [20,21]. Remarkably, Hg exposure restricted the expansion of JEG-3 trophoblastic spheroids but did not impair the secretory phenotype of decidual primary hEnSC. These results support the decreased viability, aberrant ROS production, and enhanced cell death reported herein and in previously published studies using the HTR8/SV-neo trophoblast cell line [31,45]. The antioxidant-mediated restoration of JEG-3 trophoblastic spheroid expansion in our co-culture model also corroborates the beneficial effects of antioxidants reported in trophoblast cell cultures [46,47]. Taken together, this evidence supports the notion that Hg-induced endometrial cell damage can be substantially reversed with antioxidant supplementation.

Strengths and limitations: The main strengths of this study are the use of primary human endometrial cells and Hg doses ranging from the mean baseline levels found in women’s biofluids (urine and plasma) to the maximum doses associated with initial and major signs of Hg poisoning (250–500 nM, respectively) [6,48], which were recently used by our group [18]. We recognize that while the use of primary human endometrial cells from healthy donors can faithfully replicate human endometrial features and physiology in vitro, the high intra-biopsy variability likely contributed to the large standard deviations in our experiments. While the different lifestyles, dietary habits, and environmental exposures of the participants may have contributed to the heterogeneity of endogenous Hg levels, including untreated controls in all the experiments allowed us to account for these effects. Another potential limitation is that we focused on the effect of inorganic Hg; however, other studies using different sources of Hg (as organic methylHg) reported similar cell responses and inductions of OS [15,17].

In conclusion, our study demonstrated that antioxidant supplementation can significantly reverse Hg-induced endometrial damage in primary human co-cultures of hEnEC and d-hEnSC with trophoblastic spheroids. Even though more in vitro and clinical studies are still needed, this research suggests that the use of antioxidant compounds in women attending IVF with risks of Hg exposure could improve endometrial function.

## 4. Materials and Methods

### 4.1. Study Design

**Figure 6:**
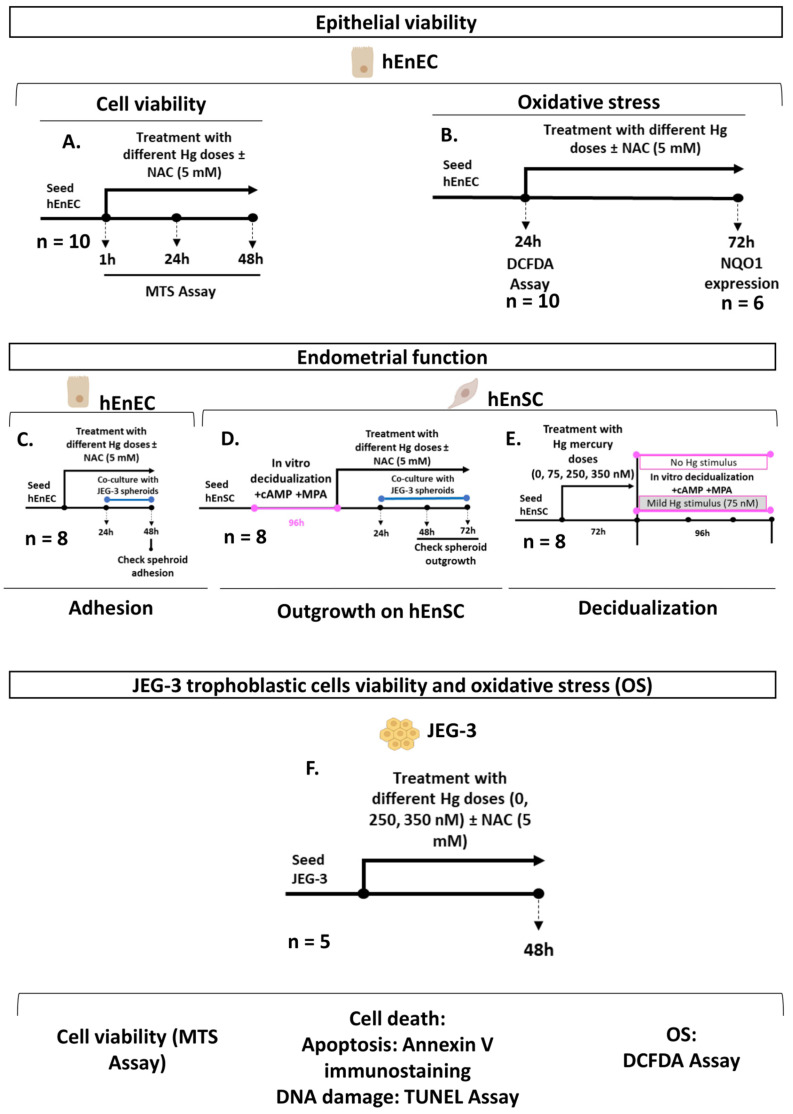
Study design. Primary human endometrial epithelial (hEnEC) and stromal cells (hEnSC) were isolated from biopsies obtained from different healthy oocyte donors (*n* = 44). (**A**) Viability of the hEnEC was measured at different timepoints using an assay. (**B**) Following 24 h of treatment with mercury (Hg) (i) 0, 4, 75, 250m or 500 nM of mercury, or (ii) 0, 250m or 350 nM of Hg alone or in combination with 5 mM N-Acetyl-L-cysteine (NAC), and the oxidative stress (OS) was evaluated through the quantification of reactive oxygen species (ROS). *NQO1* expression was evaluated in the hEnEC treated Hg alone or in combination with NAC. (**C**) Confluent monolayers of hEnEC were treated with Hg alone or in combination with NAC. The proportion of adhered JEG-3 spheroids was assessed at 48 h. (**D**) Primary decidual human endometrial stromal cells (d-hEnSC) were treated with Hg alone or in combination with NAC for 72 h. JEG-3 spheroids were added to the culture, and spheroid outgrowth was recorded. (**E**) hEnSC were pretreated with Hg prior to in vitro decidualization, and 0 or 75 nM Hg was maintained throughout the four-day decidualization regimen. The OS of the hEnSC was assessed prior to and after the decidualization process. Prolactin and the insulin-like growth factor-binding protein 1 were measured by ELISA. (**F**) JEG-3 cells were treated with Hg either with or without NAC. Then, cell viability was measured by MTS assay. Cell death was assessed by annexin V immunostaining and TUNEL assay. OS was studied by quantifying ROS (visualized with dichlorofluorescin diacetate (DCFDA) staining). cAMP, cyclic adenosine monophosphate; MPA, medroxyprogesterone acetate; *NQO1*, NAD(P)H Quinone Dehydrogenase 1. DNA, desoxyribonucleic acid.

The viability and function of the primary hEnEC and hEnSC and JEG-3 trophoblastic cells were evaluated following exposure to different doses of Hg (HgCl_2_) over 48 h. In total, 44 endometrial biopsies were allocated to the different experiments included in this research, and some biopsies were fragmented and used for different experiments. First, hEnEC viability (Figure 6A) and OS (Figure 6B) were assessed following treatment with 0, 4, 75, 250, or 500 nM Hg alone or in combination with 5 mM N-Acetyl-L-cysteine (NAC). Next, we evaluated JEG-3 trophoblastic spheroid adhesion to a monolayer of pretreated hEnEC (Figure 6C) and outgrowth into primary decidual endometrial stromal cells (d-hEnSC) (Figure 6D) under similar co-culture conditions. To characterize the effect of Hg exposure during the decidualization of primary hEnSC, hEnSC pretreated with different doses of Hg (0–350 nM) were decidualized in vitro with no (0 nM) or mild (75 nM) Hg stimulus (Figure 6E), and the efficacy of decidualization was assessed by analyzing the secretion of prolactin and insulin-like growth factor-binding protein-1 (IGFBP1) in the culture medium. Furthermore, ROS were quantified in all decidual/non-decidual, Hg-treated, and untreated hEnSC. In parallel, JEG-3 trophoblast cell viability, cell death, and ROS were evaluated following treatment with 0, 250, or 350 nM of Hg alone or in combination with 5 mM NAC (Figure 6F).

### 4.2. Participants and Biological Samples

Endometrial biopsies (*n* = 44) were collected from healthy oocyte donors *(n* = 44) at the IVI Valencia between September 2020 and December 2021. All participants met the inclusion criteria for our oocyte donation program. Main baseline characteristics are summarized in Supplementary Figure S1. Donors were fertile women (18–33 years old; body mass index (BMI) of 20–25 kg/m^2^) with normal menstrual cycles (21–35 days), and negative serologies for hepatitis B and C, human immunodeficiency virus (HIV) and syphilis. Ultrasonographic and gynecological examinations were performed on all participants to confirm normal uterine structure. Women were excluded from the study if they had an intra-uterine device (IUD) inserted, were diagnosed with a relevant pathological condition (i.e., adenomyosis, endometriosis, endometritis, pelvic inflammatory disease, abnormal bleeding, or other anatomic pathologies of the uterus), or were pregnant within three months of sample collection. Notably, we limited inter-biopsy variability by only including patients that underwent the same ovarian stimulation protocol. Endometrial biopsies were collected using a Pipelle catheter (CCD Laboratories, Paris, France) on the day of oocyte retrieval and processed on the same day to isolate the hEnEC and hEnSC.

### 4.3. Isolation and Culture of hEnSC and hEnEC

Both hEnEC and hEnSC were isolated by gravity sedimentation from endometrial biopsies, as described previously [41,49]. Briefly, human endometrial biopsies were minced into smaller pieces (≤1 mm^3^) using sterile scalpel blades, and digested with collagenase IA (C9891, Sigma-Aldrich, St. Louis, MO, USA) diluted 1:10 in phenol red-free Dulbecco’s Modified Eagle’s Medium (DMEM, D1145, Sigma-Aldrich, Madrid, Spain) overnight at 4 °C. Recovered hEnSC were resuspended in phenol red-free DMEM/nutrient mixture F12 medium (DMEM/F12, 11039047, Gibco, Thermo Fisher Scientific, Waltham, MA, USA) supplemented with 10% heat-inactivated fetal bovine serum (hiFBS; 12106C, Sigma-Aldrich, Madrid, Spain), 0.4 μg/mL amphotericin B (Gibco, 15290018, Thermo Fisher Scientific, Waltham, MA, USA), and 80 μg/mL gentamicin (Gibco, 15750045, Thermo Fisher Scientific, Waltham, MA, USA). On the other hand, the hEnEC pellets were enzymatically digested by agitation with TrypLE Select 1X (12563029, Gibco, Thermo Fisher Scientific, Waltham, MA, USA) for 5 min at 37 °C. After neutralization of the enzymatic action, the cell suspension was purified by centrifugation for 5 min at 2000 rpm, and incubated for 15 min at 37 °C in cell culture flasks with 25 cm^2^ of cell growth area (70025, SPL Life Sciences, Gyeonggi-do, Republic of Korea) containing 5 mL of DMEM/F12 supplemented with 10% hiFBS, 0.4 μg/mL amphotericin B, and 80 μg/mL gentamicin to promote adhesion of any residual stromal cells to the culture flask’s surface. The resulting supernatant was centrifuged for another 5 min at 2000 rpm to recover the purified hEnEC pellet. Culture homogeneity was assessed using morphological characteristics and verified by immunocytochemical localization of the stroma/epithelial marker vimentin and the immune cell/macrophage marker CD45 [41].

### 4.4. In Vitro Hg Exposure and Antioxidant Supplementation

Stock solutions for Hg (1 M; HgCl_2_; analytical grade, ≥98% purity, 215465, CAS 7487-94-7, Sigma-Aldrich, Madrid, Spain) and NAC (5 mM; analytical grade, ≥99% purity, A9165, CAS 616-91-1, Sigma-Aldrich, Madrid, Spain) were produced by dissolving these compounds in sterile MilliQ water (Millipore, Paris, France) and filtering before use. Working solutions of Hg and NAC were prepared by dissolving the stock solution in the corresponding cell medium supplemented with 2% hiFBS. Due to FBS containing trace levels of steroid hormones, we controlled for internal effects using the same concentration of FBS in untreated cells [41,50,51]. The Hg concentrations used in this study (i.e., 4, 25, 50, 75, 250, 350, and 500 nM) were selected based on a previous study from our group [18]. The higher doses that we assessed correspond to urinary Hg levels previously associated with renal function impairment (250 nM) and tremors induced by major Hg toxicity (500 nM) [48]. On the other hand, our 5 mM NAC treatment was adapted from the NAC dosage that was previously used alone or in combination with Hg in endometrial cell lines [14].

### 4.5. Cell Viability Assays

Cell viability of the primary hEnEC (*n* = 10) was assessed after treatment with different doses of Hg alone or in combination with 5 mM NAC. JEG-3 trophoblast cell viability (*n* = 5) was assessed after Hg treatment (0, 250, or 350 nM) alone or in combination with 5 mM NAC. Cell viability was evaluated by the mitochondrial ability to metabolize modified tetrazolium salt (MTS) in a colorimetric-based assay (CellTiter 96 Aqueous cell proliferation assay kit, G3582, Promega, Madrid, Spain). A total of 80,000 cells per well were initially seeded in 96-well culture plates. When primary cultures reached 40% confluency, cells were exposed to Hg alone or in combination with 5 mM NAC. Absorbance was measured at different timepoints in a microplate reader (SpectraMax 190, Molecular Devices, San Jose, CA, USA). Three replicates of each condition were included in each independent assay. Results are expressed as percentages of cell viability (calculated by normalizing the optical density (OD) values for each time and dose to those of control cells, which were considered to be 100% viable) ± standard deviation (SD).

### 4.6. Cell Death Assessments: Annexin V Immunostaining and TUNEL

Annexin V (ANXA V) immunostaining was performed in JEG-3 trophoblast cells (*n* = 5) following the protocol for labeling apoptotic cells for microscopy, using the Dead Cell Apoptosis Kit with annexin V FITC and Propidium Iodide for Flow Cytometry (V13242, Invitrogen, Waltham, MA, USA). Briefly, cells were fixed with 4% paraformaldehyde in PBS overnight at 4 °C, and then permeabilized in 0.2% Triton X-100 (HFH10, Invitrogen, Thermo Fisher Scientific, Waltham, MA, USA) for 15 min at room temperature. As per manufacturer instructions, cells were subsequently incubated with annexin V conjugate in 1X annexin-binding buffer (1:5) for 15 min at room temperature and washed thrice with PBS.

The TUNEL assay was performed in JEG-3 trophoblast cells (*n* = 5) using the In Situ Cell Death Detection Kit (TMR red, 12156792910, Roche, Sigma-Aldrich, Madrid, Spain), according to the manufacturer’s protocol for adherent cells. Briefly, fixed cells were permeabilized in 0.1% Triton X-100. For TUNEL-positive controls, untreated cells were incubated with 2000 U/mL DNase I in 50 mM Tris−HCl (pH 7.5) with 1 mg/mL bovine serum albumin to induce double-strand DNA breaks prior to the addition of the TUNEL reaction mixture. All samples were incubated with 150 μL of TUNEL reaction mixture (enzyme solution and label solution; 1:10), with the exception of the untreated cells, serving as the TUNEL negative- control, which were incubated exclusively with the label solution.

Following the ANXA V and TUNEL assays, cells were counterstained with DAPI (ProLong Diamond Antifade Mountant with DAPI, Molecular Probes, Life Technologies, Madrid, Spain) and visualized using an AXIO inverted fluorescence microscope (Zeiss, Jena, Germany). Three separate photomicrographs were captured per sample/condition to quantify the ANXA V- or TUNEL-positive cells and DAPI-positive nuclei. The proportions of apoptotic and DNA-damaged cells were, respectively, determined using the percentages of ANXA V- or TUNEL-positive cells to the total number of DAPI-positive cells. For each condition, the results for cell death are presented as the mean from five samples ± SD.

### 4.7. Quantification of ROS

The OS produced by Hg treatment alone, or in combination with 5 mM NAC supplementation, was measured in samples of the hEnEC (*n* = 10), hEnSC (*n* = 8) and trophoblast JEG-3 cells (*n* = 5) using a permanent fluorogenic dye to quantify cellular ROS activity (DCFDA/H2DCFDA Cellular ROS Assay Kit, ab113851, Abcam, Cambridge, UK). For this assay, cells were seeded in 96-well culture plates at 60,000 cells/well, and treated when primary cultures reached 70% confluency. While some untreated controls of each cell type were treated with an exogenous ROS inductor (tert-Butyl hydroperoxide, tBHP) to act as a positive control, others were not stained with DCFDA to act as negative controls. Three replicates of each condition were included in each independent assay. After 24 h or 48 h post-treatment, a cell-based assay for ROS detection was performed according to the manufacturer’s instructions. Photomicrographs of fluorescent DCFDA and Hoechst (94403, CAS 23491-45-4, Sigma-Aldrich, Madrid, Spain) signals were captured at 200× using an AXIO inverted microscope (Zeiss, Germany). ImageJ software (v. 1.53t) [52,53] was used for quantitative analysis of fluorescent signals. The integrated density of fluorescence (IntDen) associated with the DFCDA-positive signals indicating ROS production and Hoescht-positive signals indicating the cell nuclei were measured, respectively, in the whole photomicrograph area. The IntDen of the DCFDA-positive signal was normalized to that of the Hoescht-positive signal, and fold change (in units of fluorescence) was calculated with respect to the untreated controls. Results are presented as an overall fold change ± SD.

### 4.8. Quantification of NQO1 Gene Expression

Gene expression of *NQO1*, a gene related to antioxidant defense response, was assessed by RT-qPCR following 72 h exposure of primary hEnEC (*n* = 6) to 0, 250, or 350 nM Hg. Total RNA was isolated from each sample using the RNeasy mini kit (74536, Qiagen, Madrid, Spain), following the manufacturer’s protocol. An amount of 500 ng of total RNA was reverse transcribed into cDNA using the PrimeScript RT reagent kit (RR037B, Takara, Saint-Germain-en-Laye, France) for RT-qPCR analysis. Each 10 μL PCR reaction consisted of 10 ng of template cDNA, 5 μL of PowerUp SYBR Green Master Mix (Applied Biosystems, Madrid, Spain), 10 μM of forward and reverse primers, and nuclease-free water. The RT-qPCR was performed on a StepOnePlus RT-PCR system (Applied Biosystems, Madrid, Spain). The mRNA expression of *NQO1* was normalized to that of the internal housekeeping control (glyceraldehyde 3-phosphate dehydrogenase (*GAPDH*)), and calculated with the 2^−ΔΔCt^ method, using the untreated controls as reference. The sequences of the primers used are as follows: GAPDH (Forward 5′-3′ AGATCAAGAAGGTGGTGAAG; Reverse 3′-5′ TTGTCATACCAGGAAATGAGC) and NQO1 (Forward 5′-3′ CAGCGGCTTTGAAGAAGA; Reverse 3′-5′ GAATATCACAAGGTCTGCGG). Three technical replicates were included for each condition and target. Results are expressed as the mean fold change from six replicates ± SD.

### 4.9. In Vitro Decidualization of Primary hEnSC

Primary hEnSC were seeded in a 24-well culture plate and a short in vitro decidualization protocol (4 days) was used, as previously described [51,54,55]. Briefly, we exposed hEnSC to 1 mM medroxyprogesterone 17-acetate (MPA; M1629, CAS 71-58-9, Sigma-Aldrich, Madrid, Spain) and 0.5 mM stable cyclic adenosine 3′:5′ monophosphate analogue bromo-cyclic adenosine monophosphate (cAMP; A9501, CAS 60-92-4, Sigma-Aldrich, Madrid, Spain) in a phenol red-free DMEM/F12 supplemented with 2% hiFBS, 0.4 μg/mL amphotericin B, and 80 μg/mL gentamicin. Given the length of in vitro cultures with decidual cells and the sensitivity of primary cells to prolonged cultures, this decidualization protocol was ideal for promptly acquiring the decidual phenotype without compromising cell integrity.

At 40% confluency, primary hEnSC (*n* = 8) that were untreated or pretreated with 75, 250, or 350 nM Hg for 72 h were decidualized in vitro with no or mild (75 nM) Hg stimulus. In parallel, replicates of untreated and Hg-treated hEnSC were cultured without decidualization stimuli as negative controls (non-decidual controls; nd-hEnSC). Notably, nd-hEnSC were also grown in culture media supplemented with 2% hiFBS to avoid bias from putative trace levels of steroid hormones in hiFBS.

To assess whether antioxidant treatment (5 mM NAC) can effectively reduce Hg-induced damage in decidual hEnSC (d-hEnSC), in vitro decidualization was induced in primary hEnSC (*n* = 8). The d-hEnSC were treated with 0, 75, 250, or 350 nM Hg alone or in combination with 5 mM NAC for 72 h, and subsequently used for trophoblast outgrowth assays.

The efficacy of decidualization was evaluated by measuring secreted levels of prolactin (PRL) and insulin-like growth factor-binding protein 1 (IGFBP1) in culture media supernatants with enzyme-linked immunosorbent assays (ELISAs; NBP2-60128 (Novus Biologicals, Madrid, Spain) and IGFBP1 (KA6115, Abnova, Madrid, Spain), respectively). Detection limits for human prolactin and IGFBP1 were 2 ng/mL and <1 ng/mL, respectively. In both cases, two technical replicates of each condition were included. To account for inter-culture variability, the PRL and IGFBP1 levels in samples of each condition were normalized to those of untreated d-hEnSC. Results are presented as an overall mean percentage ± SD.

### 4.10. Culture of the JEG-3 Trophoblast Cells and Generation of Trophoblastic Spheroids

The JEG-3, derived from human placental choriocarcinoma (JEG ATCC® HTB-36™), were cultured in growth medium containing Eagle’s Minimum Essential Medium (EMEM; 670086, Gibco, Thermo Fisher Scientific, Waltham, MA, USA) supplemented with 10% hiFBS, 0.4 μg/mL amphotericin B, and 80 μg/mL gentamicin, in culture flasks with 75 cm^2^ of cell growth area (70075, SPL Life Sciences, Republic of Korea). The growth medium was replaced every three days, and cells were passaged every six days (>70% confluency). To passage JEG-3 cells, the growth medium was removed, and the flasks were washed once with 5 mL of sterile Dulbecco’s phosphate-buffered saline (PBS) with calcium and magnesium (D8662, Sigma-Aldrich, Madrid, Spain), followed by an incubation with 5 mL of TrypLE Select 1X for 5 min at 37 °C. Enzymatic action was neutralized by the addition of fresh growth medium. Following centrifugation (5 min at 2000 rpm), the supernatant was discarded, and the pellet was resuspended in 1 mL of fresh growth medium. This suspension was then seeded in a new flask with 75 cm^2^ of cell growth area and 9 mL of fresh growth medium for further culture.

JEG-3 trophoblastic spheroids were generated by seeding a 20 μL cell suspension (1:100 dilution) in ultra-low attachment 6-well culture plates (CLS3471, Corning Costar, Madrid, Spain) with 2 mL of growth medium, and culturing at 37 °C and 5% CO_2_ for 48 h. For visualization during co-culture assays, the JEG-3 trophoblastic spheroids were fluorescently labeled with CellTracker Blue CMAC (C2110, Invitrogen, Thermo Fisher Scientific, Waltham, MA, USA) diluted 1:1000 in EMEM, during a 30-min incubation at 37 °C and 5% CO_2_ in the dark. Finally, following two washes with PBS, the stained trophoblastic spheroids were co-cultured with either the hEnEC monolayer or d-hEnSC cultures, as detailed below.

### 4.11. Adhesion Assays

To evaluate trophoblast adhesion to hEnEC monolayers, primary hEnEC were initially seeded in 24-well culture plates. Primary hEnEC cultures at 40% confluency were then hormonally primed with 10 nM β-oestradiol (E2, E4389, Sigma-Aldrich, Madrid, Spain) for two days following six days of hormonal stimulation with 10 nM E2 and 1 µM of progesterone (P4, P7556, Sigma-Aldrich, Madrid, Spain) to resemble the receptive mid-secretory phase of the human menstrual cycle, in accordance with similar hormonal schemes [56]. Hormone-primed hEnEC monolayers at 80% confluency (*n* = 4) were treated with 0, 75, 250, or 350 nM Hg for 72 h. Furthermore, hEnEC (*n* = 4) were treated with 0, 250, or 350 nM of Hg alone or in combination with 5 mM NAC for 72 h. After 24 h of treatment, the JEG-3 trophoblastic spheroids were incorporated for a 48 h co-culture. Hormonal stimulation was maintained during Hg treatment and co-culture of trophoblastic spheroids. The number of spheroids attached to the hEnEC monolayer was counted after vigorously shaking culture plates and was presented as a proportion relative to the initial number of seeded spheroids. Additionally, total number of seeded and adhered spheroids was reported.

### 4.12. Outgrowth Assays

To assess trophoblast invasion in decidual stroma, primary cultures of d-hEnSC treated exclusively with 0, 75, 250, or 350 nM of Hg (*n* = 4), or 0, 250, or 350 nM of Hg alone or in combination with 5 mM NAC (*n* = 4) for 24 h were co-cultured with the JEG-3 trophoblastic spheroids for another 48 h. Trophoblast spheroid invasion was assessed by measuring trophoblastic spheroid expansion on the d-hEnSC. Photomicrographs of co-cultures were captured (Supplementary Figure S3A–C) at 400× using an AXIO inverted microscope (Zeiss, Jenda, Germany) and used to measure trophoblast outgrowth area. The integrated contouring tool in ImageJ software (v.1.53t) [52,53] was used to manually outline the perimeter of the trophoblasts and measure the outgrowth area (in µm^2^).

### 4.13. Statistical Analyses

Sample size was estimated assuming a non-directional hypothesis, a mean difference among groups of 35–40%, and a statistical power of 80%. Given these considerations, the calculated effect size was 2.25–2.5 and the estimated required sample size per experiment was 4–5 samples. Results were presented as overall means ± SD or reported as mean values with 95% confidence. Non-parametric statistics were performed because normal distribution was not assumed. Friedman’s test followed by a post hoc multiple comparison test (Dunn’s test) was used to compare percentages of cell viability obtained after MTS assays, ROS production, PRL and IGFBP1 secretion, apoptosis, adhesion percentages, and percentage of trophoblast expanded area for each condition tested. In all cases, *p*-values < 0.05 were considered statistically significant.

## Data Availability

All data presented refer to the original data generated during the study.

## Supplementary Materials

The supporting information can be downloaded at: https://www.mdpi.com/article/10.3390/ijms24108799/s1.
