# Supplementary material for: Antioxidant Supplementation Alleviates Mercury-Induced Cytotoxicity and Restores the Implantation-Related Functions of Primary Human Endometrial Cells
Source: Int J Mol Sci. 2023 May 15;24(10):8799. doi: 10.3390/ijms24108799 (PMC10218493; doi:10.3390/ijms24108799)
Supplement: Supplementary file 1 [file ijms-24-08799-s001.zip › Figure S1 figure and caption.pdf]

A **Baseline characteristics of donors.**

Characteristic	Mean ± SD	Minimum	25% percentile	75% percentile	Maximum
Race	Caucasian				
Age (years)	25 ± 4.1	18	21	28	34
BMI (kg/m^2)	22 ± 1.9	18	21	24	25
AMH (pmol/L)	25 ± 17	4.9	13	32	80
rhFSH dose / stimulation day (IU)	214 ± 50	125	180	250	302
Days of stimulation (days)	10 ± 1.5	8	9	11	13
Total rhFSH dose (IU)	2160 ± 640	1000	1675	2569	3900

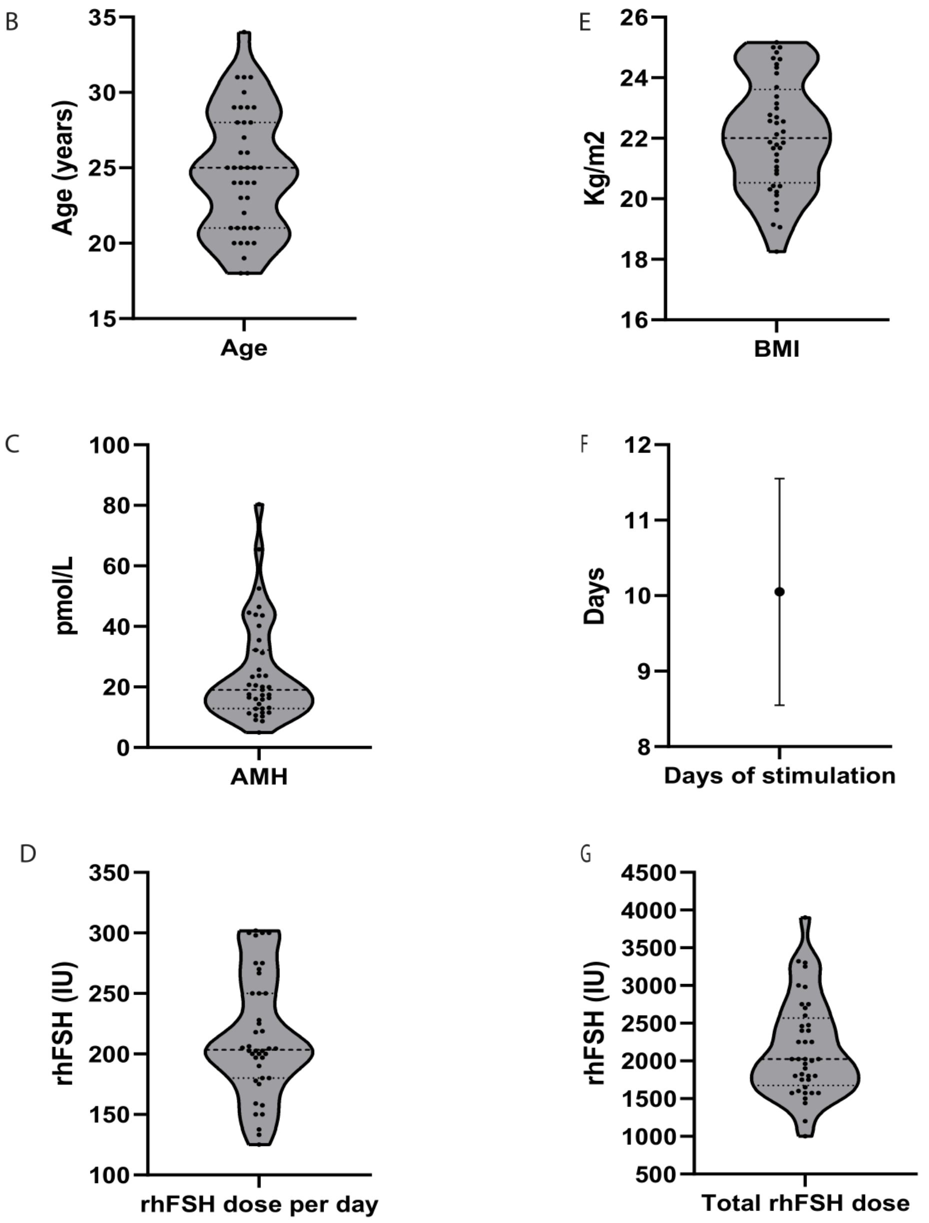

Supplementary Figure S1. Baseline characteristics of donors. (A) Descriptive statistics of donor baseline characteristics and (B-G) corresponding violin plots showing each distribution. BMI, body mass index; AMH, anti-Müllerian hormone. rhFSH, recombinant human follicle stimulating hormone.
