# Supplementary material for: Antioxidant Supplementation Alleviates Mercury-Induced Cytotoxicity and Restores the Implantation-Related Functions of Primary Human Endometrial Cells
Source: Int J Mol Sci. 2023 May 15;24(10):8799. doi: 10.3390/ijms24108799 (PMC10218493; doi:10.3390/ijms24108799)
Supplement: Supplementary file 1 [file ijms-24-08799-s001.zip › Figure S2 figure and caption.pdf]

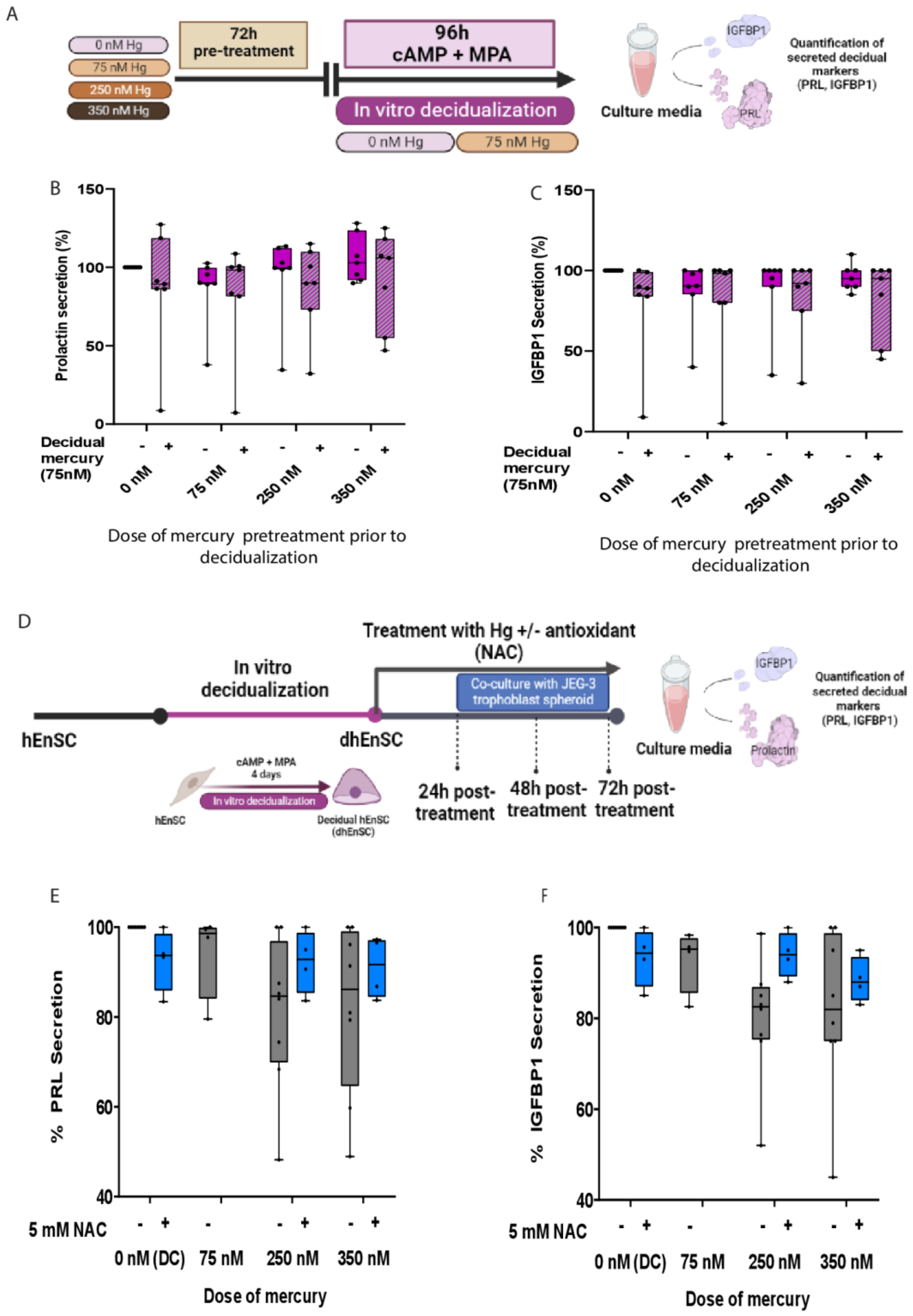

Supplementary Figure S2. Decidualization of primary human endometrial stromal cells (hEnSC) treated prior to and during in vitro dualization with Hg alone or with antioxidant compound (5 mM NAC). (A) Schematic of experimental workflow. Boxplots for the secretion of prolactin (PRL; B) and insulin-like growth factor-binding protein-1 (IGFBP1; C) from primary hEnSC pretreated with 0, 75, 250 or 350 nM for 72h and decidualized with no or mild (75 nM) Hg stimulus. (D) Schematic of experimental workflow of co-culture model of primary decidual human endometrial stromal cells (d-hEnSC) and JEG-3 spheroids. Boxplots for the secretion of prolactin (PRL; D) and insulin-like growth factor-binding protein-1 (IGFBP1; F) from d-hEnSC pretreated with 0, 250 or 350 nM either with (blue bars) or without (grey bars) 5 mM NAC for 24h and co-cultured with JEG-3 spheroids for 48h. Data displayed in boxplots (B-C, E-F) is presented as the overall mean of relative secretion measured on the final endpoint of both experiments (expressed as percentage) with respect to the untreated control (0 nM) condition ± standard deviation (SD).
