# Supplementary material for: Antioxidant Supplementation Alleviates Mercury-Induced Cytotoxicity and Restores the Implantation-Related Functions of Primary Human Endometrial Cells
Source: Int J Mol Sci. 2023 May 15;24(10):8799. doi: 10.3390/ijms24108799 (PMC10218493; doi:10.3390/ijms24108799)
Supplement: Supplementary file 1 [file ijms-24-08799-s001.zip › Figure S3 figure and caption.pdf]

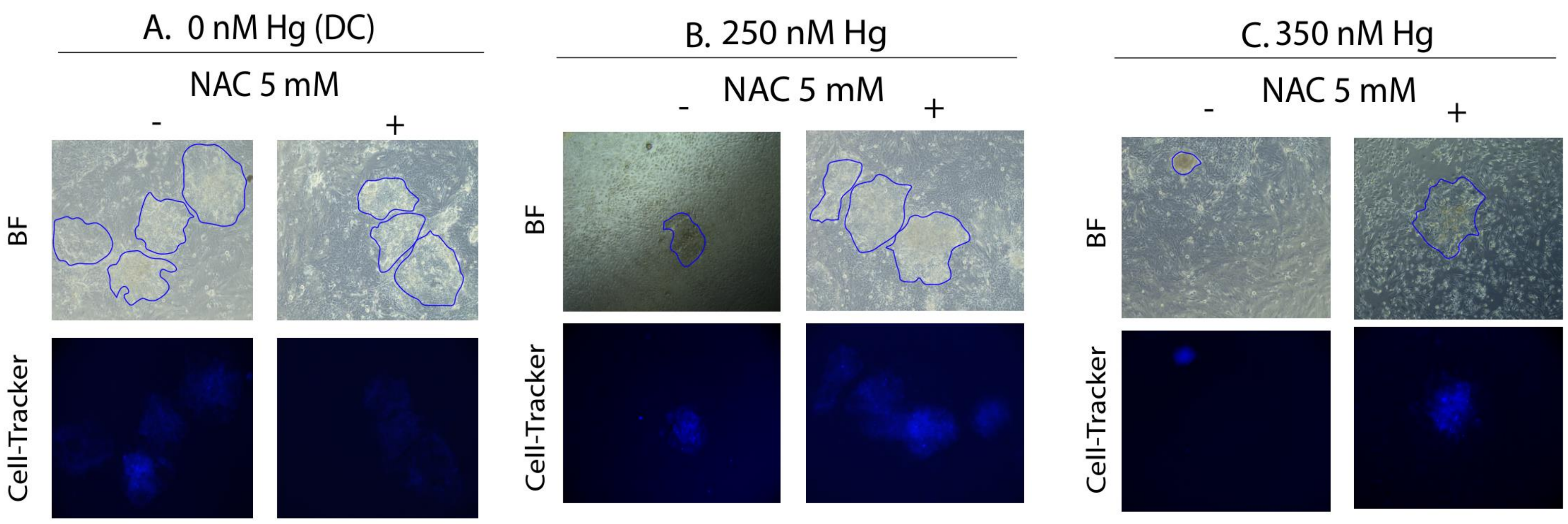


**Supplementary Figure S3.** Co-cultures of JEG-3 trophoblast spheroids and human primary decidual stromal cells treated with different Hg doses (0 nM, A; 250 nM, B and 350 nM, C) either in presence or absence of antioxidant compound (NAC 5mM). Images were captured at 400x magnification after 48h of introducing JEG-3 trophoblast spheroids in and human primary decidual stromal cultures. Bright-field (BF) and fluorescence (353 nm excitation wavelength) images were collected from each condition tested. Expanded trophoblastic outgrowth was tracked labelling JEG-3 trophoblastic spheroids with live-cell imaging probe CellTracker Blue CMAC (C2110, Invitrogen, Thermo Fisher Scientific, Waltham, MA, USA). Blue-dotted lines in BF images outline expanded trophoblastic outgrowth.
